# Chemical tools selectively target components of the PKA system

**DOI:** 10.1186/1472-6769-9-3

**Published:** 2009-02-12

**Authors:** Daniela Bertinetti, Sonja Schweinsberg, Susanne E Hanke, Frank Schwede, Oliver Bertinetti, Stephan Drewianka, Hans-Gottfried Genieser, Friedrich W Herberg

**Affiliations:** 1 Department of Biochemistry, University of Kassel, Heinrich-Plett-Str. 40, 34132 Kassel, Germany; 2 Biolog Life Science Institute, Flughafendamm 9a, P.O. Box 107125, Bremen, Germany; 3 Biaffin GmbH & Co KG, Heinrich-Plett-Str. 40, 34132 Kassel, Germany

## Abstract

**Background:**

In the eukaryotic cell the cAMP-dependent protein kinase (PKA) is a key enzyme in signal transduction and represents the main target of the second messenger cAMP. Here we describe the design, synthesis and characterisation of specifically tailored cAMP analogs which can be utilised as a tool for affinity enrichment and purification as well as for proteomics based analyses of cAMP binding proteins.

**Results:**

Two sets of chemical binders were developed based on the phosphorothioate derivatives of cAMP, Sp-cAMPS and Rp-cAMPS acting as cAMP-agonists and -antagonists, respectively. These compounds were tested via direct surface plasmon resonance (SPR) analyses for their binding properties to PKA R-subunits and holoenzyme. Furthermore, these analogs were used in an affinity purification approach to analyse their binding and elution properties for the enrichment and improvement of cAMP binding proteins exemplified by the PKA R-subunits. As determined by SPR, all tested Sp-analogs provide valuable tools for affinity chromatography. However, Sp-8-AEA-cAMPS displayed (i) superior enrichment properties while maintaining low unspecific binding to other proteins in crude cell lysates, (ii) allowing mild elution conditions and (iii) providing the capability to efficiently purify all four isoforms of active PKA R-subunit in milligram quantities within 8 h. In a chemical proteomics approach both sets of binders, Rp- and Sp-cAMPS derivatives, can be employed. Whereas Sp-8-AEA-cAMPS preferentially binds free R-subunit, Rp-AHDAA-cAMPS, displaying antagonist properties, not only binds to the free PKA R-subunits but also to the intact PKA holoenzyme both from recombinant and endogenous sources.

**Conclusion:**

In summary, all tested cAMP analogs were useful for their respective application as an affinity reagent which can enhance purification of cAMP binding proteins. Sp-8-AEA-cAMPS was considered the most efficient analog since Sp-8-AHA-cAMPS and Sp-2-AHA-cAMPS, demonstrated incomplete elution from the matrix, as well as retaining notable amounts of bound protein contaminants. Furthermore it could be demonstrated that an affinity resin based on Rp-8-AHDAA-cAMPS provides a valuable tool for chemical proteomics approaches.

## Background

The cAMP-dependent protein kinase (PKA) is a key regulator protein in eukaryotic signal transduction and is involved in several cellular processes during growth and development. Via phosphorylation of its substrate proteins PKA controls metabolic processes, cAMP mediated gene expression, cell differentiation and/or apoptosis [1]. The enzymatic activity of the PKA catalytic (C) subunit is controlled by a set of four different regulatory (R) subunit isoforms, i.e. type I and type II, both with two isoforms (α and β) each. Thus, in its inactive state PKA forms a heterotetrameric holoenzyme complex (R_2_C_2_), containing an R-subunit dimer and two C-subunit monomers. The PKA holoenzyme is activated upon cooperative binding of four molecules of the second messenger cAMP to the R-subunits, thus releasing the now active C-subunits [2]. The expression pattern of single PKA isoforms as well as the isoform specific composition of the holoenzyme and tissue specific distribution of PKA isoforms allows a tight regulation of the catalytic activity of PKA. In turn, PKA provides an important model system for kinases, allowing investigation of the molecular mechanisms of kinase function as well as the development of tools for diagnostic purposes which in turn enables their use as biomarkers [3].

Historically, purification of PKA holoenzyme from biological material was performed via anion exchange chromatography (DEAE, [4]); later, a second step based on affinity purification utilising cAMP resins was added [5,6]. However, most strategies so far resulted in either partly degraded or insoluble protein with limited yield. Therefore we set out to design novel cAMP affinity matrices for simple and rapid purification of cAMP binding proteins. These resins should provide a chemical tool that targets proteins containing the conserved cAMP binding domains, specifically PKA R-subunits while fulfilling the following criteria required for efficient affinity binders:

1. Purify high quantities of selected protein of interest;

2. yield functionally active protein;

3. provide a purification procedure with mild but efficient elution conditions while retaining high yields of protein;

4. obtain nucleotide-free proteins which can easily be used for further interaction studies and biochemical assays;

5. provide an easy-to-use procedure applicable in chemical proteomics.

In addition to cAMP's role as a general activator of all holoenzyme isoforms, with PKA representing the main intracellular effector of cAMP, there are still other targets of cAMP like nucleotide-gated ion channels [7], cAMP degrading phosphodiesterases [8] or Epac, the cAMP-regulated guanine nucleotide-exchange factor for Rap 1 and 2 [9], all containing one or more highly conserved cyclic nucleotide binding (CNB) domains [10].

**Figure 1:**
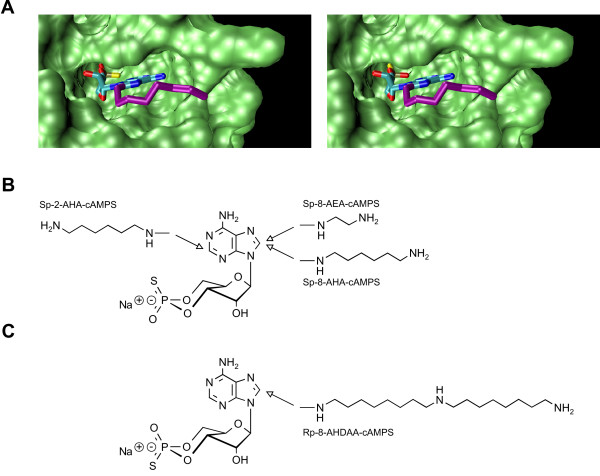
**Chemical structures of cyclic nucleotides**. (A) Model of the bovine RIα subunit (PDB-ID 1RGS) with bound agonist (Sp-8-AHA-cAMPS, left) and antagonist (Rp-8-AHA-cAMPS, right). The figure was created using standard settings in VMD 1.8.4 [58]. Chemical structures of Sp-cAMPS (B) and Rp-cAMPS (C) analogs. Arrows indicate positions of attached linkers.

PKA R-subunits and specific components of the cAMP signalling network can be precisely targeted from complex protein mixtures using highly specific cAMP analogs covalently coupled to agarose beads. Generally, two groups of chemical tools are used for the affinity purification of functional complexes: agonist and antagonist binders (Fig. 1A). Agonist binders are synthetic cyclic nucleotides which bind the R-subunits that are complexed with interaction partners (e.g. AKAPs), however, these agonists cause the PKA holoenzyme to dissociate. Antagonist binders, interacting preferentially with the intact, non dissociated PKA holoenzyme, can be utilised to identify interaction partners that bind to the entire complex (R_2_C_2_), including proteins interacting with the C-subunits.

In the present study several cyclic nucleotide analogs were synthesised and optimised for their binding properties to the respective components of the PKA system using an iterative approach based on direct SPR binding studies. Subsequently, these cAMP analogs were coupled to a solid support and tested for binding and elution in a one step purification procedure probing all four recombinantly expressed R-subunit isoforms. Furthermore, we tested optimised cyclic nucleotides analogs for their application in a chemical proteomics approach addressing either the R-subunits or the intact PKA holoenzyme complex along with physiological interaction partners derived from animal tissue.

## Results

In previous studies, hundreds of cyclic nucleotides were developed and characterised regarding their binding ability to proteins containing cyclic nucleotide binding (CNB) domains [11-14].

### Improved cAMP analogs as novel tools for PKA R-subunit purification

For our developments we used optimised binders based on Sp-cAMPS (Fig. 1A left panel, 1B), a cAMP analog where the axial exocyclic oxygen atom of the cyclic phosphate is replaced by a sulphur atom, resulting in an approximately 10-fold reduced affinity towards the R-subunit when compared to cAMP alone [15-17]. In order to couple Sp-cAMPS to a solid phase, analogs with different spacers in positions 2 or 8 of the adenine base (Sp-8-AEA-cAMPS, Sp-8-AHA-cAMPS, Sp-2-AHA-cAMPS, Fig. 1B) were designed and synthesised. Two complementary strategies were combined for the characterisation of improved binders for PKA R-subunit purification. In initial kinetic studies, surface plasmon resonance (SPR) was employed and selected cAMP analogs were immobilised on a sensor chip and the association and dissociation patterns of all four R-subunit isoforms were analysed in detail. In an additional approach, these cAMP analogs were coupled to agarose beads and their ability for affinity purification of R-subunits was studied via pull down experiments. In both sets of experiments, 8-AHA-cAMP and 8-AEA-cAMP were used as controls, since they represent conventional cAMP analogs which have been commonly applied for purification of PKA R-subunits or for interaction studies in previous reports [6,12,18-29].

### SPR binding studies

**Figure 2:**
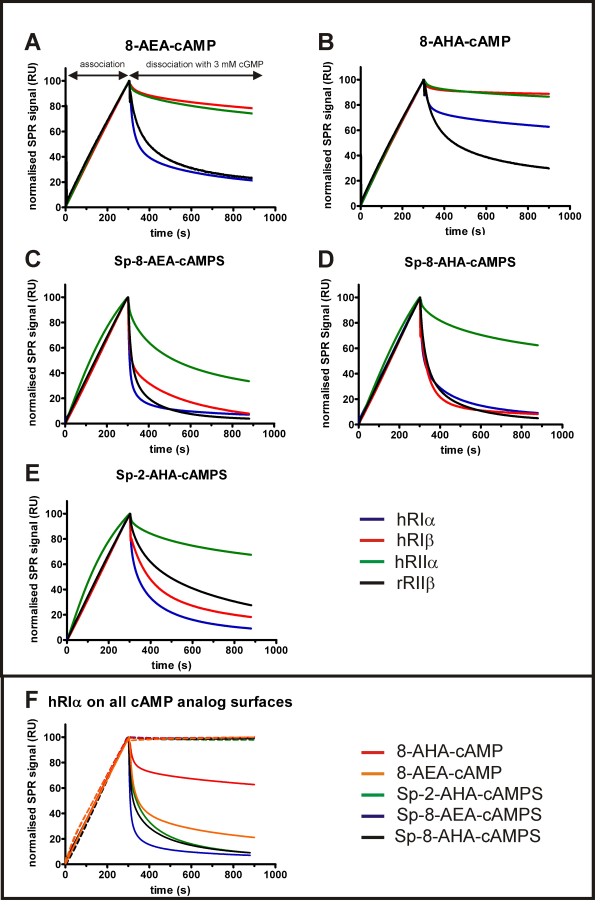
**Interaction of R-subunit isoforms with immobilised cAMP analogs investigated by SPR**. Binding of all four R-subunit isoforms (100 nM) to immobilised 8-AEA-cAMP (A), 8-AHA-cAMP (B), Sp-8-AEA-cAMPS (C) and Sp-8-AHA-cAMPS (D) Sp-2-AHA-cAMPS (E) using a flow rate of 10 μL/min in buffer A containing 0.005% P20. Association and dissociation of hRIα (blue), hRIβ (red), hRIIα (green) and rRIIβ (black) were monitored for 5 and 10 min, respectively. The dissociation was monitored in the presence of 3 mM cGMP. (F) hRIα binding to five different cAMP analogs. 100 nM hRIα was injected to immobilised 8-AHA-cAMP (red), 8-AEA-cAMP (orange), Sp-2-AHA-cAMPS (green), Sp-8-AEA-cAMPS (blue) and Sp-8-AHA-cAMPS (black). Dissociation was initiated either by buffer A containing 0.005% P20 (dotted lines) or by 3 mM cGMP in the same buffer (solid lines). Experimental setup was performed as described above.

8-AEA-cAMP, 8-AHA-cAMP, Sp-8-AEA-cAMPS, Sp-8-AHA-cAMPS and Sp-2-AHA-cAMPS were covalently coupled to a sensor surface using NHS/EDC chemistry and the association and dissociation patterns of the four different R-subunit isoforms (RIα, RIβ, RIIα, RIIβ) were analysed using a Biacore 2000 instrument (Fig. 2). All four R-subunit isoforms showed fast association behaviour when binding to the immobilised cAMP analogs. No notable dissociation could be detected when switching the instrument to buffer, thus indicating a stable interaction between the R-subunit and the immobilised cAMP analogs (Fig. 2F). When adding cGMP (3 mM) during the dissociation phase the dissociation behaviour from an immobilised cyclic nucleotide analog was affected by (i) the exchange of the exocyclic oxygen to a sulphur in the immobilised cAMP analog, (ii) the chosen linker of the cAMP analog and (iii) the R-subunit isoform. In general, dissociation in the presence of cGMP was slower for conventional cAMP analogs compared to the newly designed Sp-cAMPS analogs. When comparing dissociation from all Sp-cAMPS analogs, a slower dissociation was observed from the 2-substituted analog (Fig. 2E) than from the 8-substituted analog (Fig. 2C, D). Among the R-subunit isoforms, RIIα displayed the slowest dissociation from all five analog surfaces tested.

Generally, fast binding to the affinity matrix as well as rapid and complete dissociation under elution conditions are preferred for purification, without the dissociation of protein from the affinity matrix under washing conditions. From the observed elution patterns, Sp-8-AEA-cAMPS was considered the best candidate for efficient elution of hRIα (Fig. 2C, F), especially when compared to 8-AHA-cAMP and 8-AEA-cAMP which conventionally have been employed (Fig. 2A, B) [18,27-29]. Panels A and B (Fig. 2) clearly demonstrate that the RIβ and RIIα dissociate rather slow from the non Sp-cAMPS analogs, even in the presence of cGMP. In general, for the R-subunit isoforms RIβ, RIIα and RIIβ, the dissociation with cGMP is fast and almost identical for the 8-substituted cAMPS analogs.

As a consequence of the high surface ligand density the association and dissociation patterns of the R-subunits to the immobilised cAMP analogs were severely affected by mass transfer limitations. These limitations are due to depletion of the analyte (here the R-subunits) being in close proximity to the sensor surface during association, thus prohibiting the quantitative analyses of association and dissociation kinetics. Furthermore, rebinding effects in the dissociation phase are intensified by the presence of four cAMP binding sites per R-subunit dimer [30-32]. Still, the high surface density of cAMP analogs on the sensor surface very well represents the situation that is occurring on the agarose beads during binding and elution.

### Purification of the R-subunit isoforms using Sp-cAMPS agaroses

**Figure 3:**
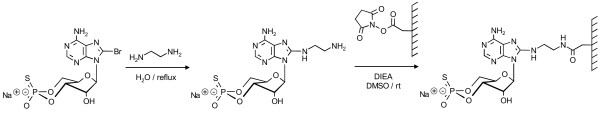
**Coupling of cyclic nucleotides to a solid support**. Representative scheme showing synthesis and coupling of Sp-8-AEA-cAMPS to NHS-activated agarose beads (Affi-Gel^® ^10, BIO-RAD). For details of synthesis and coupling see Methods section.

**Figure 4:**
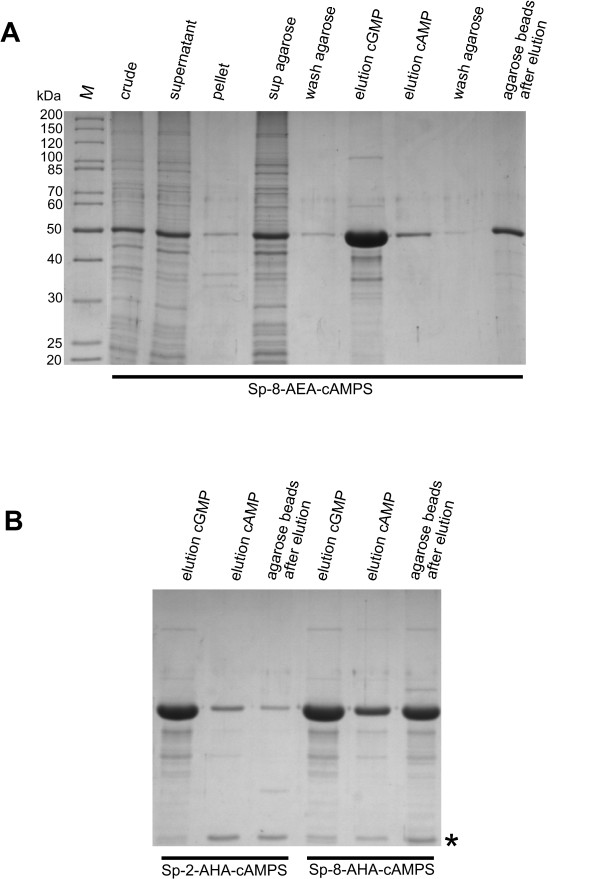
**Purification of hRIα subunit with different Sp-cAMPS analog agaroses analysed by SDS-PAGE**. (A) *crude*: total cell extract of *E. coli *BL21 DE3 Codonplus RIL with overexpressed hRIα at 52 kDa; *supernatant*: soluble fraction of bacterial lysate after French Press and centrifugation; *pellet*: insoluble fraction; *sup agarose*: unbound protein after incubation with Sp-8-AEA-cAMPS agarose; *wash agarose*: aliquot after 6 washing steps with buffer B; *elution cGMP *and *elution cAMP*: elution with 10 mM cGMP and subsequently with 10 mM cAMP, respectively; *wash agarose*: aliquot after 6 washing steps with buffer B; *agarose beads after elution*: remaining protein on the agarose beads after the two elution and washing steps. *M*: molecular weight marker (Page-Ruler Unstained Protein Ladder, Fermentas). (B) Total cell extract of *E. coli *BL21 DE3 Codonplus RIL with overexpressed hRIα was incubated with Sp-2-AHA-cAMPS and Sp-8-AHA-cAMPS, respectively, and eluted with cGMP (10 mM, lane 1 and 4) and subsequently with cAMP (10 mM, lane 2 and 5). Only a low amount of protein was left on Sp-2-AHA-cAMPS agarose after elution with cAMP (lane 3), whereas significant amounts of R-subunit remained on Sp-8-AHA-cAMPS agarose (lane 6). Asterisk indicates Chloramphenicol Acetyltransferase from *E. coli *BL21 (DE3) Codon Plus RIL as identified by MS.

The analogs tested in SPR (8-AEA-cAMP, 8-AHA-cAMP, Sp-8-AEA-cAMPS, Sp-8-AHA-cAMPS and Sp-2-AHA-cAMPS) were coupled to agarose beads using NHS chemistry (Fig. 3). Bacterial cell lysate containing overexpressed RIα was incubated with each analog coupled to agarose (a representative purification procedure with Sp-8-AEA-cAMPS is shown in Fig. 4A). After several washing steps were performed, no notable dissociation of the RIα subunit from the agarose was detected. Elution of the protein with 10 mM cGMP resulted in a protein fraction containing highly pure and active R-subunit as determined by SDS-PAGE and spectrophotometric activity assay [33], yielding 12 mg protein per approximately 400 μL of agarose slurry. Only little additional protein could be eluted subsequently with cAMP, still leaving some protein on the agarose beads (Fig. 4A).

cGMP elution from the other two agaroses (Sp-8-AHA-cAMPS and Sp-2-AHA-cAMPS) resulted in half the amount of purified PKA R-subunit (7.2 mg protein per approximately 400 μL of agarose slurry, Fig. 4B) when compared to the yield from the Sp-8-AEA-cAMPS agarose. Furthermore, hRIα could only be partially eluted from the Sp-8-AHA-cAMPS agarose with 10 mM cAMP, while leaving a significant amount of protein on the beads, thus indicating that RIα binds too tight to this analog and therefore can not be used for this affinity purification procedure. Additionally, both the Sp-2-AHA-cAMPS and Sp-8-AHA-cAMPS agarose showed a 25 kDa protein coeluted with cGMP, it was identified by mass spectrometry (MS) as *E. coli *Chloramphenicol Acetyltransferase which is abundant in the BL21 (DE3) Codon Plus RIL strain (Fig. 4B).

Thus, from the three affinity resins tested, only Sp-8-AEA-cAMPS fulfils and exceeds all necessary requirements for improved affinity purification when compared to conventional 8-AEA- or 8-AHA-cAMP agarose chromatography [18] and thus appears to be the best option for isolating large quantities of pure and active RIα.

### Comparison of conventional agaroses 8-AHA-cAMP and 8-AEA-cAMP with Sp-8-AEA-cAMPS

**Figure 5:**
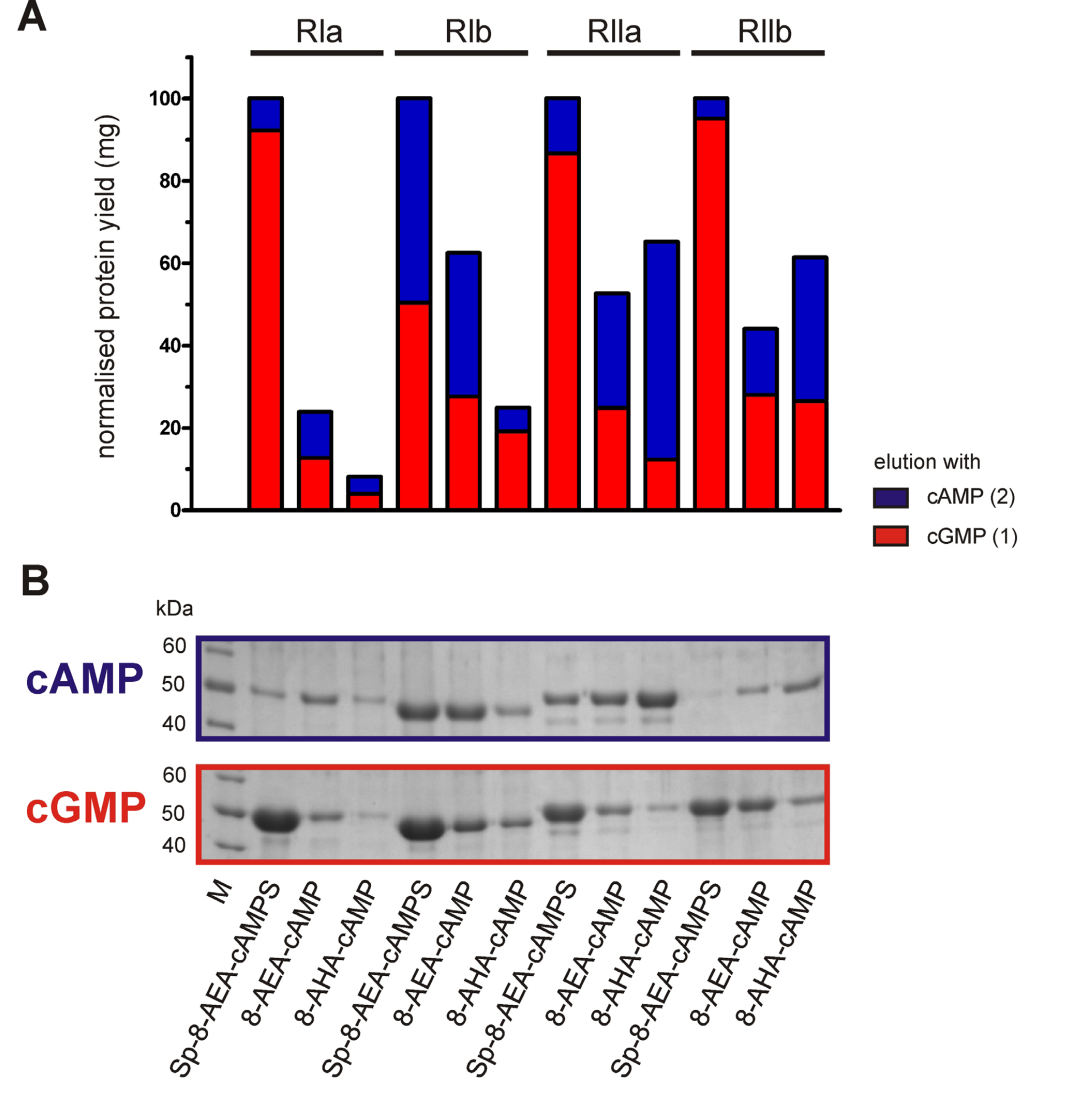
**Improvements on cAMP analogs for the purification of all R-subunit isoforms**. Comparison of the protein yield eluted from the agarose with cGMP (red bars) followed by cAMP (blue bars) indicated on the plot. Data were normalised to the total protein yield from both elution steps using Sp-8-AEA-cAMPS agarose as determined by BCA assay. The lower panel shows a SDS-PAGE of samples from each elution fraction (equal volumes of original fractions, cGMP elution red framed, cAMP elution blue framed). *M*: molecular weight marker (Page-Ruler Unstained Protein Ladder, Fermentas).

The Sp-8-AEA-cAMPS resin as well as the conventional agaroses 8-AHA-cAMP and 8-AEA-cAMP were all tested via small scale purification with all four R-subunit isoforms, namely RIα, RIβ, RIIα and RIIβ. The protein yields eluted first with cGMP and then subsequently in a second elution step with cAMP are displayed in Fig. 5. As a control the cAMP elution was performed according to Diller *et al. *[18]. All R-subunit isoforms were obtained in a pure and active form as determined by a spectrophotometric assay [33]. Since the highest overall yield of the respective R-subunit was obtained using Sp-8-AEA-cAMPS agarose, this amount was set as the 100% reference point (Fig. 5A). Interestingly, mild elution of RIα subunit with cGMP from the Sp-8-AEA-cAMPS agarose is nine times more efficient than the cGMP elution from the 8-AEA-cAMP agarose. Furthermore, a fourfold higher yield was obtained from the cGMP elution only using the Sp-cAMPS agarose than from combining both elution steps (cGMP and cAMP) employing 8-AEA-cAMP together. Purification of RIα with 8-AHA-cAMP agarose was even less efficient than using 8-AEA-cAMP (Fig. 5B). Still, the amount of RIβ, RIIα and RIIβ isoforms eluted from our newly designed Sp-8-AEA-cAMPS agarose was a minimum of 40% greater than from the conventional agaroses.

In a further experiment all four R-subunit isoforms were purified with the Sp-8-AEA-cAMPS agarose (data not shown). The purification of RIα and RIIα isoforms resulted in 12 mg protein per liter expression media. For RIβ and RIIβ isoforms 5 mg and 3 mg protein were purified respectively. All affinity purified proteins were verified by MS (data not shown). The entire purification procedure of each R-subunit isoform was completed within 8 h.

### Binding of the PKA holoenzyme complex to agonist and antagonist

Based on the purification strategy described above, novel cAMP derivatives with antagonist properties were designed to analyse the PKA holoenzyme (R_2_C_2_) complex and target physiological interaction partners via a chemical proteomics approach. Only cAMP analogs derived from the lead structure Rp-cAMPS (replacement of the equatorial exocyclic oxygen of the cyclic phosphate, Fig. 1A right, 1C) have been shown to act as PKA-antagonists [17,34-36], whereas the Sp-cAMPS analogs and cAMP itself act as agonists (Fig. 1A left, 1B). The general assumption that Rp-cAMPS binds to the R-subunit in the intact PKA holoenzyme thus preventing complex dissociation [37] was addressed by a combination of SPR binding analysis and Mass spectrometry (BIA-MS).

**Figure 6:**
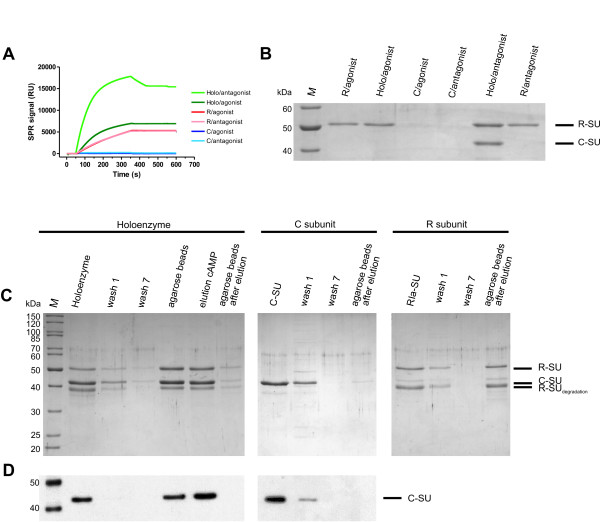
**Rp-cAMPS analogs bind the intact PKA holoenzyme complex**. The entire PKA type I holoenzyme complex (R_2_C_2_) binds highly specific to Rp-8-AHDAA-cAMPS as demonstrated by SPR (A), BIA MS (B) as well as affinity purification followed by SDS-PAGE (C) and Western blot analysis (D). (A) SPR binding pattern of type I holoenzyme, hRIα and Cα (as indicated, 250 nM each) on antagonist (Rp-8-AHDAA-cAMPS) and agonist (8-AHA-cAMP) surfaces. Measurements were performed in buffer A containing 0.005% P20, 1 mM ATP and 10 mM MgCl_2 _at a flow rate of 5 μL/min. (B) BIA-MS: SDS-PAGE analysis of protein recovered from the experiments depicted in (A). Type I holoenzyme, R-subunit and C-subunit were injected to the agonist and antagonist surfaces as described and were eluted with 0.2% SDS after a 4 min wash step. The eluted material from 15 repetitive runs was pooled and analysed by SDS-PAGE, MS and Western blot analysis (not shown). C-subunit (42 kDa) and R-subunit hRIα (50 kDa) are indicated. *M*: molecular weight marker (Page-Ruler, unstained protein ladder, Fermentas). (C) Binding of PKA type I holoenzyme complex (R_2_C_2_, left panel), free C-subunit (center panel) and free R-subunit (right panel) to antagonist agarose (Rp-8-AHA-cAMPS, 600 pmoles, for chemical structure see Fig. 1C) determined with SDS-PAGE. (D) Immunoblot analysis of SDS gels from panel (C) using anti-C-subunit antibody.

Therefore the antagonist Rp-8-AHDAA-cAMPS (for chemical structure see Fig. 1C) and the agonist 8-AHA-cAMP were covalently coupled to two sensor surfaces on a single CM5 sensor chip. 250 nM of each, PKA holoenzyme (R_2_C_2_), free R-subunit and C-subunit were injected to both sensor surfaces. Fig. 6A shows the resulting binding curves for type I holoenzyme. Maximum binding signals of 17,000 and 6,900 RU on the antagonist and on the agonist surfaces were observed, respectively. These difference in the binding signals indicates that the entire holoenzyme complex is captured to the antagonist surface whereas on the agonist surface only the free R-subunit is bound, since the immobilised agonist causes holoenzyme dissociation and release of the C-subunit. Hence, these differences in binding signals reflect the mass difference between the holoenzyme complex (R_2_C_2_) and the free R-subunit (R_2_). A control experiment was performed, injecting free R-subunit over both surfaces and similar binding signals of approximately 5,300 RU each were obtained for the agonist as well for the antagonist surface (Fig. 6A). Furthermore, binding signals to the agonist surface using free R-subunit and the PKA holoenzyme complex were in the same range, again indicating that the holoenzyme dissociates when interacting with the agonist surface. As an additional control C-subunit alone was injected over both analog surfaces. No binding was observed, demonstrating that the C-subunit does not contribute to the binding signal.

To prove that the intact holoenzyme complex was bound to the antagonist sensor surface a BIA MS experiment was performed (Fig. 6A, B). 250 nM of each, PKA holoenzyme (R_2_C_2_), free R-subunit and C-subunit were injected and after a 4 min wash, the sensor surface was incubated with 0.2% SDS. Proteins bound to the nucleotide surface were eluted using the microrecovery function (Biacore 3000 Control Software 4.1) and the content of 15 subsequent elutions was subjected to SDS-PAGE (Fig. 6B), displaying two distinct bands. These bands correspond to R- and C-subunits, as identified by LC-ESI MS/MS and Western blot analysis (data not shown). Both, R- and C-subunit, were detected in equal amounts according to Coomassie staining (Fig. 6B), strongly supporting the hypothesis that functionally intact holoenzyme complex (R_2_C_2_) was bound to the antagonist surface and subsequently eluted. For a control the same amount of holoenzyme was also injected to the agonist surface and analysed as described above. Only R-subunit, but no C-subunit, could be detected by SDS-PAGE (Fig. 6B), MS and Western blot analysis (data not shown).

Based on the BIA-MS results (Fig. 6B), the antagonist was coupled to agarose beads via NHS chemistry (Fig. 3). These beads were then incubated with purified holoenzyme complex. Samples of each step of the pull down experiment were analysed via a SDS-PAGE (Fig. 6C) as well as analysed by Western blot with an anti-C-subunit antibody (Fig. 6D), proving that Rp-cAMPS derivatised agarose provides an extremely valuable tool for holoenzyme purification. An additional control was performed with free R- and C-subunit. Both were incubated with the antagonist beads and then analysed with SDS-PAGE. Only the R-subunit but not C-subunit was detected verifying the BIA-MS results.

### Targeting the PKA interactome from pig brain using agonist and antagonist agaroses

**Figure 7:**
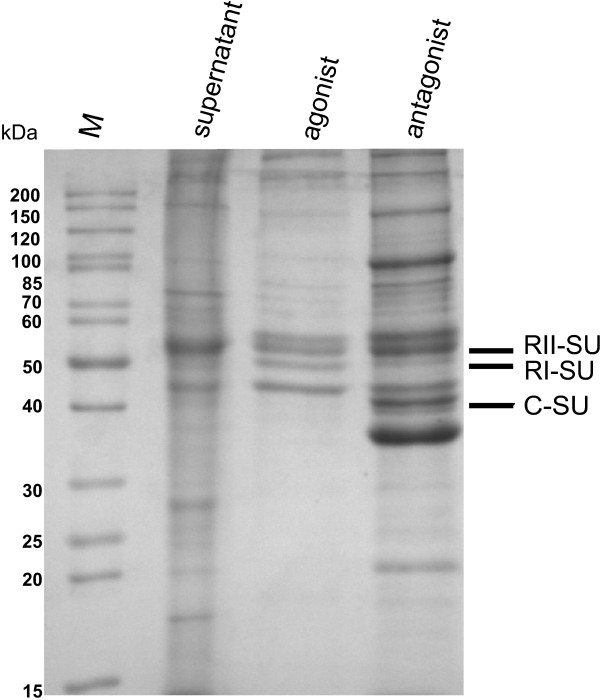
**Affinity enrichment of components of the cAMP-pathway using agonist and antagonist agaroses**. Proteins from pig brain tissue were enriched using agonist (Sp-8-AEA-cAMPS) and antagonist (Rp-8-AHDAA-cAMPS) agaroses and subsequently eluted with cAMP. Fractions were separated on a 12% SDS-PAGE. *supernatant*: soluble protein fraction of pig brain after lysis, filtration and centrifugation; *agonist/antagonist*: protein eluted from the agonist/antagonist agarose with 25 mM cAMP. C-subunits (42 kDa), R-subunits RI (50 kDa) and RII (52 kDa) subunits are indicated, each identified by MS (data not shown). *M*: molecular weight marker (Page-Ruler, unstained protein ladder, Fermentas).

In a proof of principle experiment agonist (Sp-8-AEA-cAMPS) and antagonist agarose (Rp-8-AHDAA-cAMPS) were incubated with pig brain lysate in order to investigate, if endogenously expressed R-subunit and PKA holoenzyme can be pulled out of biological material. After incubation of the soluble protein fraction with the affinity resins, the beads were washed six times and the bound proteins were subsequently eluted with 20 mM cAMP and analysed by SDS-PAGE (Fig. 7).

Using the agonist as affinity reagent, all four R-subunit isoforms were pulled from pig brain lysate. When using the antagonist, additionally all major C-subunit isoforms (Cα, Cβ and Cγ; Fig. 7) were identified based on MS (data not shown). In addition to the PKA subunits other distinct sets of proteins were pulled out from pig brain tissue using either agonist or antagonist agaroses (Bertinetti *et al. *in preparation). This indicates, that functional complexes of cAMP interactome can be pulled out of tissue lysates with these new chemical tools, proving the opportunity for the discovery of additional components of the cAMP signalling pathway.

## Discussion

During the last 30 years several approaches have been used to selectively purify fully functional R-subunit isoforms of PKA, either endogenous from animal tissues [6] or recombinantly expressed in bacteria [38]. Most purification strategies have been based on ion exchange chromatography. There were also early attempts to use cAMP analogs linked via the N6 or C8 position of the adenine ring to an agarose matrix for affinity purification of R-subunits from several tissues [5]. In most cases, however, the affinity of the R-subunit to the various cAMP analogs used was too high to subsequently and successfully elute the protein efficiently. Only under harsh conditions could the R-subunit be removed from the matrix either by one of the two procedures: 7 M urea [6,39] or cAMP elution at elevated temperatures [23,38]; while still yielding not only less protein than using ion exchange chromatography but also protein that was insoluble and/or proteolytically degraded [26]. In order to increase the yield of nucleotide-free and highly active R-subunit attempts to use fusion tags for purification [40-42]. However, fusion tags to the N-terminus as well as to the C-terminus of the R-subunit turned out to be problematic. Modifications of the N-terminus can interfere with R-subunit dimerisation and binding of A kinase anchoring proteins [43]. Since fusion tags on the C-terminus are located in close proximity to the cAMP binding domains, the cyclic nucleotide binding properties can be affected. Cleavage of the tag is an additional purification step which may lead to a loss of protein as well as cause additional proteolytic products.

We synthesised 3 different Sp-cAMPS analogs (Sp-8-AEA-cAMPS, Sp-8-AHA-cAMPS, Sp-2-AHA-cAMPS) and characterised the binding to all four R-subunit isoforms in direct interactions studies using SPR. Subsequently these agonists were coupled to solid supports and used for affinity purification of recombinantly expressed R-subunits. All three analogs displayed superior purification strategies when compared to conventional cAMP analogs (8-AEA-cAMP, 8-AHA-cAMP).

Using Sp-8-AEA-cAMPS agarose it is now possible to obtain large yields of active and nucleotide-free R-subunit without the use of denaturants. This is especially important since remaining nucleotide in the CNB domain would interfere with subsequent studies. Furthermore decreased stability of Urea unfolded and refolded protein occurs [44].

In general, affinity reagents, addressing a specific subset of proteins or protein complexes, are of growing importance for many applications in proteomics. This includes enrichment of proteins or subcellular components as well as removal of unwanted cell debris subcellular components, proteins and metabolites. Therefore, our described cAMP analogs not only provide a highly selective tool as an affinity material for purification of PKA R-subunits, but can also serve as a tool that particularly targets the cAMP sub-proteome (Bertinetti *et al. *in preparation). Here we can demonstrate that components of cAMP signalling pathways were selectively complexed with their physiological interaction partners thus demonstrating that phosphorothioate cAMP analog agaroses are extremely valuable for comparable chemical proteomics studies. Furthermore, phosphorothioate cAMP analogs provide an additional advantage as they were shown to be highly stable against phosphodiesterase activity [45] and are therefore well suited for use with cellular lysates.

In a recent approach, Scholten et al. [24] used conventional cyclic nucleotide agaroses to pull out components of the cGMP/cAMP interactome from rat heart tissue in order to identify cyclic nucleotide binding proteins and their interaction partners. Since elution with cAMP from the 8-AEA-cAMP agarose was not sufficient for subsequent MS analysis, those studies had to be performed with material that was obtained by boiling the incubated agarose beads in SDS loading buffer. With the newly designed Sp- and Rp-cAMPS agaroses described here, specific binding and elution of cAMP binding proteins and a reduction of false positives can be achieved since elution with cAMP can be considered as an additional purification step. The use of these novel tools now enables us to also pull out interaction partners of intact holoenzyme, which is especially important for identification of novel interaction partners of the C-subunits of PKA such as the recently described A-kinase interacting proteins (AKIPs, [46]) in the holoenzyme form.

## Conclusion

Novel cAMP analogs based on phosphorothioate were optimised in an iterative approach combining design, synthesis and direct SPR binding studies. After coupling to a solid support, two sets of chemical binders were tested for highly efficient purification procedure of all isoforms of PKA R-subunit. Sp-8-AEA-cAMPS was identified as the most valuable tool for purification purposes, yielding milligram amounts of both highly pure and active R-subunit within 8 h. Furthermore, Sp- and Rp-cAMPS derived analogs have the potential to be employed in a chemical proteomics approach targeting distinct cAMP sub-proteomes.

## Methods

### Chemicals

All chemicals used were of the purest grade available and were obtained either from Sigma-Aldrich (Seelze), Roth (Karlsruhe) or Applichem (Darmstadt). The following cAMP-analogs were synthesised by Biolog LSI (Bremen): 8-AHA-cAMP, 8-AEA-cAMP, Sp-cAMPS, Sp-8-AHA-cAMPS, Sp-2-AHA-cAMPS, Sp-8-AEA-cAMPS, Rp-cAMPS, Rp-8-AHA-cAMPS, Rp-8-AHDAA-cAMPS. A representative scheme with synthesis and coupling of Sp-8-AEA-cAMPS to agarose beads is provided in Fig. 3.

### Synthesis of cyclic nucleotide derivatives and coupling to agarose

All cAMP- and ω-aminoalkyl-substituted Sp-cAMPS analogs (for chemical structure see Fig. 1B) were synthesised as described [22,47] with some minor modifications. Typically, 100 μmoles of (Sp)-8-Br-cAMP(S), (Sp)-2-Cl-cAMP(S) or (Rp)-8-Br-cAMP(S) (Biolog) and 1,000 μmoles of 1,ω-diaminoalkan (Fluka) were dissolved in 10 mL water and refluxed until no starting material was detectable by HPLC analysis (first step in the reaction pathway Fig. 3). The reaction mixtures were neutralised with diluted HCl, concentrated by rotary evaporation under reduced pressure, and subsequently purified by means of semi-preparative reversed phase HPLC (YMC ODS-A 120–11, YMC). The column was washed with 100 mM NaH_2_PO_4_, pH 7, followed by water. Each cAMP(S) analog was eluted with a gradient from 100% water to 100% acetonitrile. The product containing fractions were collected and evaporated under reduced pressure to obtain 8-AHA-cAMP, 8-AEA-cAMP, Sp-8-AHA-cAMPS, Sp-8-AEA-cAMPS, Sp-2-AHA-cAMPS and Rp-8-AHDAA-cAMPS in yields of 60–80% with purities > 99% (by HPLC). The structure of each cAMP(S) analog was confirmed by UV/VIS spectrometry and FAB/MS or ESI/MS analysis.

cAMP(S) analogs were coupled to NHS-activated agarose beads (Affi-Gel^® ^10, BIO-RAD) according to the manufacturer's instructions (Fig. 3). Briefly, 6.6 μmoles of cAMP(S) analog and 7.26 μmoles ethyldiisopropylamine were added per mL settled gel, suspended in DMSO. Reaction mixture was carefully shaken for 2–18 h at ambient temperature until no further consumption of starting material was detectable by analytical HPLC monitoring. Any unreacted NHS-groups of the agarose gels were blocked by addition of 20 μmoles ethanolamine per mL settled gel by incubation for 1 h. After filtration and multiple washing with subsequently 2 × 25 mL 20% ethanol, 2 × 25 mL H_2_O and 2 × 25 mL 30 mM NaH_2_PO_4_, pH 7, each agarose gel was stored in 30 mM NaH_2_PO_4_, 1% NaN_3_, pH 7 at 4°C. Ligand densities were 6 μmoles/mL 8-AHA-cAMP, 6 μmoles/mL 8-AEA-cAMP, 4 μmoles/mL Sp-8-AEA-cAMPS agarose, 5 μmoles/mL Sp-8-AHA-cAMPS agarose, 6 μmoles/mL Sp-2-AHA-cAMPS agarose and 6 μmoles/mL Rp-8-AHDAA-cAMPS agarose.

### Direct binding studies of Sp-cAMPS analogs using SPR

Sp-2-AHA-cAMPS, Sp-8-AEA-cAMPS, Sp-8-AHA-cAMPS (for chemical structures see Fig. 1B), 8-AHA-cAMP and 8-AEA-cAMP were dissolved in 100 mM HEPES-KOH pH 8 by cautious heating (max. 70°C) and filtered. The concentrations of the stock solutions were determined via their respective extinction coefficient. CM5 sensor chip surfaces (research grade, Biacore AB) were activated for 10 min with NHS/EDC according to the manufacturer's instructions (amine coupling kit, Biacore AB). The analogs (3 mM) were injected for 7 min (running buffer: 100 mM HEPES, pH 8). Deactivation of the surface was performed with 1 M ethanolamine-HCl, pH 8.5. Each flow cell was activated, coupled and deactivated individually with a flow rate of 5 μL/min at 20°C. A reference cell (Flow cell 1) was activated and deactivated without ligand immobilisation.

All interaction analyses were performed at 20°C in 150 mM NaCl, 20 mM MOPS, pH 7 (buffer A) containing 0.005% (v/v) surfactant P20, using a Biacore 2000 instrument (Biacore AB). Binding analyses were performed by injection of 100 nM hRIα, hRIβ, hRIIα and rRIIβ (all proteins were purified classically by DEAE cellulose chromatography [48]) at a flow rate of 10 μL/min. Association and dissociation were monitored for 5 min and 10 min, respectively. Dissociation was performed in buffer A containing 0.005% P20 in the presence or absence of 3 mM cGMP. The sensor surfaces were regenerated after each binding cycle by two short injections of 3 M guanidinium HCl. After subtracting the reference cell signal, binding data were normalised (Fig. 2).

### Purification of hRIα using Sp-cAMPS agaroses

Bacterial cells overexpressing R-subunit were lysed using a French Pressure Cell (Thermo Electron) in lysis buffer containing 20 mM MOPS pH 7, 100 mM NaCl, 1 mM β-mercaptoethanol, 2 mM EDTA and 2 mM EGTA (buffer A). The crude lysate was centrifuged at 27,000 *g *for 30 min at 4 C. Three different Sp-cAMPS agaroses (Sp-8-AHA-cAMPS, Sp-8-AEA-cAMPS and Sp-2-AHA-cAMPS agarose) were tested side by side in a one step purification strategy. 1.2 μmoles of coupled analog were used for each purification, corresponding to approximately 400 μL of agarose slurry. 12 mL supernatant from 500 mL bacterial culture were incubated with the respective affinity matrices. Binding was carried out in a batch format by gently rotating over night at 4°C. After washing the agarose seven times with 1.25 mL lysis buffer each, the protein was eluted with 1.25 mL of 10 mM cGMP in buffer B (buffer A plus 1 mM β-mercaptoethanol) by gentle rotation at 4°C for 1 h followed by an elution using 10 mM cAMP in buffer B instead of cGMP. Excess of nucleotide was removed using a PD10 gel filtration column (Amersham Pharmacia). cGMP bound to the cyclic nucleotide binding pockets was removed by extensive dialysis against buffer B.

The purification strategy of RIβ with Sp-8-AEA-cAMPS follows in principle the procedure described for RIα.

### Purification of type II R-subunits using Sp-8-AEA-cAMPS agarose

The purification strategy of RII isoforms follows the procedure described for hRIα except cell lysis was performed in buffer containing 20 mM MES pH 6.5, 100 mM NaCl, 5 mM EDTA, 5 mM EGTA and 5 mM β-mercaptoethanol (buffer C) including the protease inhibitors Leupeptin (0,025 mg/100 mL, Biomol), TPCK and TLCK (each 1 mg/100 mL, Biomol). After cell lysis, the soluble protein fraction was incubated in a batch format with Sp-8-AEA-cAMPS agarose (1.4 μmol analog). The agarose was washed twice with 10 mL buffer D (20 mM MES pH 7, 1 M NaCl, 5 mM β-mercaptoethanol) and subsequently with buffer C containing protease inhibitors. Two elution steps were performed with 1 mL 25 mM cGMP in buffer C and exchanged to 20 mM MES pH 6.5, 150 mM NaCl, 2 mM EDTA, 2 mM EGTA and 1 mM β-mercaptoethanol using a PD10 column (Amersham).

### Compared purification of all R-subunits using different cAMP analog agaroses

The side by side comparison of Sp-8-AEA-cAMPS, 8-AHA-cAMP and 8-AEA-cAMP for purification was performed as described for the RI and RII purification. Crude lysate from one litre expression culture was divided into three equal aliquots and incubated in a small scale experiment with 100 μL of the respective agaroses. The cGMP elution was followed by a second elution step with 40 mM cAMP at room temperature for 30 minutes according to [18].

### BIA-MS

Rp-8-AHDAA-cAMPS (for chemical structure see Fig. 1C) and 8-AHA-cAMP were dissolved by cautious heating (max. 60°C) in 100 mM Borate pH 8.5 with 20% DMSO and 100 mM HEPES pH 8, respectively, filtered and coupled to the sensor surface as described above. All microrecovery experiments were performed in buffer A containing 10 mM MgCl_2_, 1 mM ATP with 0.005% (v/v) surfactant P20 on a Biacore 3000 instrument. 250 nM PKA type I holoenzyme (R_2_C_2_), free RIα subunit or C-subunit were injected over the analog sensor surfaces in separate experiments. The association phase was monitored for 5 min followed by a 4 min washing step with running buffer. Bound protein was eluted by incubating the sensor surface with 0.2% SDS for 90 s and the eluted material was recovered in a capped vial. The sensor surfaces were regenerated by three subsequent injections of 3 M guanidinium HCl.

The recovered material from 15 repetitive cycles was pooled and applied to a 12% SDS-PAGE [49] (Fig. 6B) for protein identification via MS.

### Fishing of PKA holoenzyme complex with Rp-cAMPS agarose

Purified recombinant mCα, hRIα and PKA holoenzyme were each incubated with Rp-8-AHDAA-cAMPS agarose (600 pmoles coupled analog, for chemical structure see Fig. 1C) for 2 h at 4°C. The agarose was washed seven times with 1 mL buffer E (buffer B containing 1 mM ATP and 10 mM MgCl_2_). Protein was eluted with 1 mL 20 mM cAMP in buffer E by gentle rotation at room temperature for 1 h. The entire supernatant of each step was precipitated with TCA and applied to SDS-PAGE (Fig. 6C). For Western blotting, the samples were transferred to PVDF membrane and immunoblotted with anti-PKA C-subunit antibody (Santa Cruz Biotechnology, PKAα cat C-20) visualised by enhanced chemiluminescence (Fig. 6D).

### Fishing of PKA holoenzyme complex with agonist and antagonist agarose from pig brain lysate

Fresh pig brain tissue was homogenised in buffer F (buffer E in the presence of protease inhibitors (complete, EDTA free, Roche), 2 mM NADH and 20 mM sucrose). After centrifugation at 13,700 g for 25 min, the supernatant was filtered and incubated with 150 μL agarose (corresponding to 1 μmole of Sp-8-AEA-cAMPS or Rp-8-AHDAA-cAMPS) over night at 4°C. The beads were washed six times with 1.5 mL buffer F. Elution was carried out with 1 mL of buffer E containing 20 mM cAMP by gentle rotation at room temperature for 1 h. All samples were precipitated with TCA for SDS-PAGE (Fig. 7).

### Biochemical characterisation of the proteins

The purification of the R-subunits was analysed by 12% SDS-PAGE [49] unless otherwise noted and proteins were stained with colloidal Coomassie Brilliant Blue dye modified after Neuhoff *et al. *[50-52]. After electrophoresis, remaining SDS was removed by heating and rinsing in distilled water. Overnight staining with 0.1% Coomassie^® ^Brilliant Blue G 250 in 5% aluminium sulphate octadecahydrate and 2% phosphoric acid resulted in intense blue bands with low background (Fig. 4, 5, 6, 7). Protein concentration was determined by a colorimetric assay using BSA as a standard [53]. The biological activity of the proteins was verified by a spectrophotometric phosphotransferase assay using the substrate peptide Kemptide (LRRASLG, Biosynthan) according to Cook *et al. *[33].

### Mass spectrometry analysis

Protein bands were excised from one-dimensional SDS-PAGE [49] and digested in gel with trypsin according to published procedures [54], modified by omitting all prewashing steps. After equilibrating in water, the gel pieces (Fig. 4, 6B, 7) were excised and homogenised via centrifugation (1,6000 g) through 10 μL pipet tips (MBP) and collected in small reaction vials (CS-Chromatographie Service). Destaining, reduction and alkylation were omitted and 25 μL digestion buffer containing 50 mM NH_4_HCO_3_, 100 ng/μL of trypsin (sequencing grade, Promega) were added directly to the gel slurry and incubated at 50°C for a minimum 1 h [55]. After a short centrifugation the supernatants were diluted with 40 μL of 0.3% formic acid for analysis in a nanoLC-ESI-MS/MS (nanoLC-Ultimate HPLC-system, LC Packings, Dionex coupled online to a linear ion trap mass spectrometer 4000 QTRAP™, Applied Biosystems), as described in [56,57]. The MS/MS spectra were searched against a nonredundant sequence database (MSDB) using MASCOT (Matrix Science), version 1.9.05. Taxonomy was restricted to mammals with variable modifications on deamidation (NQ), myristoylation (N-Term. G), oxidation (M) and phosphorylation (ST).

## Authors' contributions

DB, SS and SEH performed the expression and purification of the recombinant proteins as well as the biochemical characterisation and the chemical proteomics experiments. FS and HGG synthesised and purified the cyclic nucleotide analogs and performed coupling to the agarose. SD carried out the SPR measurements, OB the mass spectrometry analyses, SS the BIA-MS experiments and DB, SS and FWH wrote the manuscript and prepared the figures.
